# Lymphocyte subsets in hemophilic patients with hepatitis C virus infection with or without human immunodeficiency virus co-infection: a nested cross-sectional study

**DOI:** 10.1186/1471-2326-5-2

**Published:** 2005-01-18

**Authors:** Mingdong Zhang, Thomas R O'Brien, William C Kopp, James J Goedert, for the Multicenter Hemophilia Cohort Study

**Affiliations:** 1 Viral Epidemiology Branch, Division of Cancer Epidemiology and Genetics, National Cancer Institute, Rockville, MD; USA; 2 SAIC Frederick, Inc., Frederick, MD, USA; 3 Other collaborators and institutions of the Multicenter Hemophilia Cohort Study are in the Appendix

## Abstract

**Background:**

With chronic infection, hepatitis C virus (HCV) RNA can be detected in B cells and associated with B-cell disorders, but these are not well defined.

**Methods:**

The relationship between HCV infection and lymphocyte subpopulations was evaluated rigorously in 120 asymptomatic hemophilic patients, randomly selected from a prospective cohort study. CD4^+ ^T cells, CD8^+ ^T cells, CD19^+ ^B cells, and CD56^+ ^NK cells were quantified by flow cytometry using cryopreserved peripheral blood mononuclear cells of 24 hemophilic patients in each of five age-matched groups [uninfected; chronic HCV with or without human immunodeficiency virus (HIV); and cleared HCV with or without HIV].

**Results:**

As expected, patients with HIV had significantly reduced CD4^+ ^and increased CD8^+ ^T cells. Irrespective of HIV, patients with chronic HCV infection had approximately 25% fewer CD19^+ ^B cells than those without chronic HCV infection.

**Conclusions:**

These data support the hypothesis that asymptomatic patients with chronic HCV infection have an altered B-lymphocyte population.

## Background

The natural history of hepatic C virus (HCV) infection may include B-cell diseases. HCV can be detected in peripheral blood mononuclear cells (PBMCs) and may also replicate in these cells [1-3]. Type II cryogloblinemia and some non-Hodgkin lymphomas have been linked to HCV [4], but links between B cells and asymptomatic HCV infection are poorly defined.

One study reported that hemophilic patients co-infected with HCV and human immunodeficiency virus (HIV) had reduced B cells and CD4^+ ^T cells [5]. To further characterize PBMC immunophenotypes in patients with chronic or cleared HCV infection, we quantified proportions of CD4^+ ^T cells, CD8^+ ^T cells, B cells, and natural killer (NK) cells in PBMCs of carefully selected, well characterized hemophilic patients.

## Methods

### Study subjects

Patients with hemophilia or another congenital coagulation disorder (herein referred to as "hemophilia") enrolled in the Multicenter Hemophilia Cohort Study [6,7] were categorized into five groups: no HIV or HCV infection (HIV-/HCV-); chronic (viremic) HCV infection without HIV (HIV-/HCV RNA+); chronic (viremic) HCV infection with HIV (HIV+/HCV RNA+); cleared (non-viremic) HCV infection without HIV (HIV-/HCV RNA-); and cleared (non-viremic) HCV infection with HIV (HIV+/HCV RNA-). From each of the five categories, 24 age-matched subjects were randomly selected. Non-viremic subjects were considered to have cleared HCV spontaneously, as they were selected from among all those who, without specific therapy, were negative for HCV RNA in two specimens collected at least 6 months apart. Viremic subjects were selected from among all those with an HCV RNA level above the 67^th ^percentile of all HCV-positive MHCS patients (described below), all of whom had received HCV-contaminated plasma products many years earlier and thus were considered to have chronic HCV infection. All samples were taken before 1996, the year when highly active antiretroviral therapy (HAART) became available for treatment of HIV infection.

### HCV and HIV assays

HIV status was defined by antibody testing with licensed immunoassays and immunoblot confirmation. HCV antibody status was determined with a commercially available second- or third-generation enzyme immunoassay, with most of the reactive samples confirmed by recombinant immunoblot assay (HCV RIBA2.0 or 3.0, Chiron Corp., Emeryville CA). HCV RNA was detected and viral load was determined with branched-DNA technology [Quantiplex HCV RNA 2.0 Assay (bDNA), Chiron Corp., Emeryville CA] with a lower limit of sensitivity of 200,000 genome equivalents/mL, which is 31,746 international units (IU)/mL. HIV-1 viral load was determined with the HIV Amplicor Monitor (Roche Molecular Diagnostics, Branchburg, NJ).

### Flow cytometry

A vial of 5 million cryopreserved PBMCs, stored in vapor phase LN_2 _until testing, was analyzed by flow cytometry for proportions of CD4^+^, CD8^+^, CD19^+^, and CD56^+ ^cells. In a biological safety cabinet, vials were thawed in a 37°C water bath with agitation. To each, 300 units of DNase I (Rnase-free, Roche Molecular Biochemicals) was added. The contents were transferred to a 15 ml polypropylene tube. Thawed cells were slowly diluted with RPMI-1640 media supplemented with 20% fetal calf serum, mixed frequently, then pelleted at 1000 rpm in a Sorvall RT6000 refrigerated centrifuge. The liquid was decanted. Cells were mixed in residual volume by gentle shaking, washed a second time with 4.0 ml of thawing media, then resuspended in 2.0 ml calcium- and magnesium-free Dulbecco's phosphate buffered saline (PBS) containing 4% heat-inactivated human AB serum to block high affinity Fc receptors. After 10 minutes the cells were pelleted in the centrifuge, then resuspended in 0.6 ml of PBS/2% BSA. 100 μl of cell suspension was added to each of five 12 mm × 75 m polypropylene tubes containing three-color combinations of fluorescently tagged monoclonal antibodies (CD45/CD14/CD3, CD45/CD4/CD3, CD45/CD8/CD3, CD45/CD19/CD3 and CD45/CD56/CD3). The lymphocyte gate was defined with light scatter properties and CD45/CD14/CD3. A Coulter XL flow analyzer was used with stops set to collect 5,000 CD3^+ ^lymphocytes in each sample, if possible. Listmode files were analyzed offline using Coulter System II software (version 3.0). Quality control criteria included purity of gated lymphocytes; percentage recovery of lymphocytes in the gate; reproducibility of CD3 between tubes (range <4%); and sum of CD3^+^, CD3^-^/CD19^+^, CD3^-^/CD56^+ ^(range 90–110%).

### Statistical analysis

The primary analysis was performed on proportions, rather than absolute levels, of each PBMC subset, to reduce variance that results from counting total lymphocytes in peripheral blood [8]. The Kruskal-Wallis and Wilcoxon rank sum tests were used to compare the lymphocyte subsets between groups, particularly chronic versus cleared HCV. Statistical analyses were done with the Statistical Analysis System version 6.0 (Cary, NC).

## Results

### Characteristics of study subjects

Of the 120 patients, 78 had hemophilia A (factor VIII deficiency). Because patients with hemophilia A generally required more intensive clotting factor replacement therapy as well as product (Factor VIII concentrate) that was more infectious for HIV than was Factor IX concentrate used for patients with hemophilia B, hemophilia A was significantly more common in the two groups with HIV co-infection (n = 19 with chronic HCV and n = 21 with cleared HCV) than the three groups without HIV co-infection (n = 11 to 14, p = 0.01). By design, the five groups had similar ages (mean 26.5 years, range 3.4 – 54.8 years, Table 1). HCV RNA levels were higher with HIV co-infection (median 4.6 × 10^6 ^IU/mL) than with HCV only (median 2.4 × 10^6 ^IU/mL, P < 0.01). Among 48 HIV-infected subjects, 6 (25%) with cleared and 7 (29%) with chronic HCV had clinically defined AIDS (P = 0.75) when their PBMCs were collected for this study. HIV viral load did not differ between the nine with cleared and the eight with chronic HCV who were tested on the date PBMCs were isolated (P = 0.89). Ten of 120 subjects were chronically infected with hepatitis B virus, and the prevalence of chronic HBV infection did not differ among 5 groups (P = 0.12). Only two subjects, both HIV-infected but one with cleared HCV infection and one with chronic HCV infection, had developed ascites, a manifestation of hepatic decompensation, at the time that their PBMCs were collected for this study.

**Table 1:** Age and peripheral blood mononuclear cell subsets (median, interquartile range) by viral infection status.

	Viral Infection Status^a^					
Age and Cell Type	HIV- HCV-	HIV+ HCV+ HCV RNA-	HIV+ HCV+ HCV RNA+	HIV- HCV+ HCV RNA-	HIV- HCV+ HCV RNA+	P^b^

	(n = 24)	(n = 24)	(n = 24)	(n = 24)	(n = 24)	

Age (years)	24.4 (10.8–38.7)	26.7 (18.6–34.0)	25.6 (20.9–31.0)	22.5 (15.8–30.4)	30.0 (17.0–34.7)	0.80
CD4^+ ^(%)	40.4 (35.5–49.2)	12.4 (2.7–27.0)	15.3 (2.3–24.4)	40.1 (34.4–45.7)	41.4 (36.2–45.8)	<0.0001
CD8^+ ^(%)	26.3 (24.4–33.0)	52.3 (42.6–59.5)	55.1 (49.2–62.6)	26.0 (20.4–32.8)	29.1 (22.3–37.6)	<0.0001
CD19^+ ^B (%)	15.5 (12.6–21.7)	15.4 (10.5–22.2)	11.4 (4.7–15.7)	17.8 (13.7–22.2)	13.3 (10.7–19.5)	0.04
CD56^+ ^NK (%)	9.0 (6.8–12.9)	7.0 (3.7–9.7)	6.5 (4.1–13.8)	9.7 (6.7–13.5)	8.4 (6.5–12.4)	0.22

^a ^HIV, human immunodeficiency virus; HCV, hepatitis C virus; RNA, presence or absence of HCV viremia, as detected by branched DNA assay (median HCV viral loads 4.6 × 10^6 ^and 2.4 × 10^6 ^IU/mL in HIV+ and HIV- subjects, respectively).

^b ^ANOVA test for comparison of age and Kruskal-Wallis test for comparisons of cell proportions.

### CD4^+ ^T cells

As expected, subjects with HIV had lower proportions of CD4^+ ^T cells whether they had cleared HCV (12.4% vs. 40.1%) or chronic HCV (15.3% vs. 41.4%, P#0.0001; Table 1). Patients with cleared versus chronic HCV, however, had similar proportions of CD4^+ ^T cells, regardless of HIV status (P∃0.82).

### CD8^+ ^T cells

CD8^+ ^T cells differences mirrored those of CD4^+ ^T cells (Table 1). Subjects with HIV had higher proportions of CD8^+ ^T cells whether they had cleared HCV (52.3% vs. 26.0%) or chronic HCV (55.1% vs. 29.1%, P#0.0001). Regardless of HIV, subjects with cleared and with chronic HCV had similar proportions of CD8^+ ^T cells (P∃0.30).

### CD19^+ ^B cells

Proportions of CD19^+ ^B cells differed significantly among the five groups (Kruskal-Wallis P = 0.04; Table 1). In pairwise comparisons, CD19^+ ^B cells were significantly lower with HIV and chronic HCV infections compared to uninfected subjects (11.4% vs.15.5%, P = 0.03). CD19^+ ^B cells also were lower, albeit not significantly, with chronic HCV than with cleared HCV infection in both HIV-uninfected (13.3% vs. 17.8%, P = 0.09) and HIV-coinfected (11.4% vs. 15.4%, P = 0.08) groups. CD19^+ ^B-cell levels did not differ between uninfected subjects and those with cleared HCV infection (P = 0.63).

CD19^+ ^B-cell proportions did not correlate with HCV viral load (Spearman R = 0.06, P = 0.78).

### CD56^+ ^NK cells

Proportions of CD56^+ ^NK cells did not differ significantly among the five groups (Kruskal-Wallis P = 0.22; Table 1), suggesting that HCV infection status of hemophilia patients had limited effect on CD56^+ ^NK cells.

## Discussion

Among HIV-infected subjects, we found that CD19^+ ^B-cell proportion was statistically significantly lower, from 15.5% to 11.4%, with chronic HCV infection, a fractional reduction of one-quarter [(15.5–11.4)/15.5 = 0.26]. We found subjects with chronic HCV without HIV co-infection had a reduction of the same magnitude, albeit of marginal statistical significance. HIV infection was more frequent with hemophilia A than with other coagulation disorders [6], but this did not confound the association of HCV chronicity with lower B-cell proportions. In addition, we observed an approximately one-quarter reduction (25%) in estimated absolute B-cell count in chronic HCV, despite higher variance (data not presented) [8]. Overall, our results corroborate and are of similar magnitude to those reported from Japan by Yokozaki et al [5].

Infection of B cells by HCV is controversial. Both B cells and hepatocytes express CD81, a possible receptor for HCV [9]. If HCV can replicate in B cells, as implied by detection of negative-strand RNA [10,11], then one might postulate HCV-related pathogenesis or apoptosis, as observed in B-cell lines [12]. B-lymphocytopenia also could occur during initial HCV infection and contribute to HCV chronicity by impairing neutralizing antibody response to HCV envelope 2 (E2) protein's hypervariable region-1 [13].

We found no differences in proportions of CD56^+ ^NK cells with HCV infection, but this does not negate possible impairment of NK cell function with HCV infection. *In vitro*, binding of HCV E2 to CD81 inhibits the functions of cross-linked NK cells [14,15]. CD56^+ ^NK cell proportion was reduced among our subjects who cleared HCV despite HIV co-infection, an observation that has no ready explanation and may have appeared by chance.

Our study is limited by its cross-sectional design and analysis of PBMCs collected long after primary HCV and HIV infections had occurred. We cannot define sequential PBMC changes in relation to critical virologic events, such as spontaneous clearance of HCV. Furthermore, the cells that we detected in cryopreserved PBMCs may not be representative of those in fresh PBMCs. Still, we did observe the expected associations of CD4^+ ^and CD8^+ ^T cells with HIV infection. These findings, our careful flow cytometry methods, and our rigorous study design, with frequency matching on age and random selection of subjects from a large, well characterized cohort, probably offer a reliable "snapshot" of PBMCs in hemophilic patients infected with HCV alone or co-infected with HIV.

## Conclusions

We found no association of cleared or chronic HCV infection with altered levels of CD4^+ ^or CD8^+ ^T cells, although this does not negate the likely functional importance of HCV-specific T-cell subpopulations in clearance of HCV [16,17]. Subjects with high level, chronic HCV viremia, but not those with cleared HCV infection, had reduced CD19^+ ^B-cell levels. Further studies need to clarify the temporal sequence of B-cell changes with HCV chronicity and associated clinical conditions.

## Appendix: Collaborating investigators (and institutions) in the Multicenter Hemophilia Cohort Study

M. Zhang, J.J. Goedert, T.R. O'Brien, P.S. Rosenberg, C.S. Rabkin, E.A. Engels, D. Whitby (National Cancer Institute, Rockville and Frederick MD); M.E. Eyster (Division of Hematology and Oncology, Pennsylvania State University Medical Center, Hershey PA); B. Konkle (Cardeza Foundation Hemophilia Center, Philadelphia PA); M. Manco-Johnson (Mountain States Regional Hemophilia and Thrombosis Program, University of Colorado, Aurora CO); D. DiMichele, M.W. Hilgartner (Hemophilia Treatment Center, New York Presbyterian Hospital, New York NY); P. Blatt (Christiana Hospital, Newark DE); L.M. Aledort, S. Seremetes (Hemophilia Center, Mount Sinai Medical Center, New York NY); K. Hoots (Gulf States Hemophilia Center, University of Texas at Houston, Houston TX); A.L. Angiolillo, N.L.C. Luban (Hemophilia Center, Children's Hospital National Medical Center, Washington DC); A.Cohen, C.S. Manno (Hemophilia Center, Children's Hospital of Philadelphia, Philadelphia PA); C. Leissinger (Tulane University Medical School, New Orleans LA); G.C. White II (Comprehensive Hemophilia Center, University of North Carolina, Chapel Hill NC); M.M. Lederman, S. Purvis, J. Salkowitz (Case Western Reserve University School of Medicine, Cleveland OH); C.M. Kessler (Georgetown University Medical Center, Washington DC); A. Karafoulidou, T. Mandalaki (Hemophilia Center, Second Regional Blood Transfusion Center, Laikon General Hospital, Athens, Greece); A. Hatzakis, G. Touloumi (National Retrovirus Reference Center, Athens University Medical School, Athens, Greece); W. Schramm, F. Rommel (Medizinische Klinik Innerstadt der Maximilian, Universitaet Muenchen, Munich, Germany); P. de Moerloose (Haemostasis Unit, Hôpital Cantonal Universitaire, Geneva, Switzerland); S. Eichinger (University of Vienna Medical School, Vienna, Austria); K.E. Sherman (University of Cincinnati Medical Center, Cincinnati OH); and B.L. Kroner (Research Triangle Institute, Rockville MD).

## Disclaimer

"The content of this publication does not necessarily reflect the views or policies of the Department of Health and Human Services, nor does mention of trade names, commercial products, or organization imply endorsement by the U.S. Government."

## Competing interests

The author(s) declare that they have no competing interests.

## Authors' contributions

MZ and JG conceived of the study and drafted the manuscript. MZ performed the random sampling and statistical analyses. JG provided advice on the sampling and statistical analyses, obtained funding, managed the parent cohort, and supervised the virologic testing. TOB provided advice on the design. WK provided advice on and performed the FACS analyses. All authors read and approved the final manuscript.

## Pre-publication history

The pre-publication history for this paper can be accessed here:


